# Sex Dimorphism of Allergen-Induced Secreted Proteins in Murine and Human Lungs

**DOI:** 10.3389/fimmu.2022.923986

**Published:** 2022-06-28

**Authors:** Mahadevappa Hemshekhar, Dina H. D. Mostafa, Victor Spicer, Hadeesha Piyadasa, Danay Maestre-Batlle, Anette K. Bolling, Andrew J. Halayko, Christopher Carlsten, Neeloffer Mookherjee

**Affiliations:** ^1^Manitoba Centre for Proteomics and Systems Biology, Department of Internal Medicine, University of Manitoba, Winnipeg, MB, Canada; ^2^Department of Immunology, University of Manitoba, Winnipeg, MB, Canada; ^3^Department of Pathology, School of Medicine, Stanford University, Palo Alto, CA, United States; ^4^Air Pollution Exposure Laboratory, Division of Respiratory Medicine, Department of Medicine, University of British Columbia, Vancouver, BC, Canada; ^5^Department of Air Quality and Noise, Norwegian Institute of Public Health, Oslo, Norway; ^6^Department of Physiology and Pathophysiology, University of Manitoba, Winnipeg, MB, Canada; ^7^Biology of Breathing Group, The Children’s Hospital Research Institute of Manitoba, Winnipeg, MB, Canada

**Keywords:** inflammation, allergen, asthma, biological sex, house dust mite (HDM), eosinophil peroxidase (EPO), lung

## Abstract

Biological sex influences disease severity, prevalence and response to therapy in allergic asthma. However, allergen-mediated sex-specific changes in lung protein biomarkers remain undefined. Here, we report sex-related differences in specific proteins secreted in the lungs of both mice and humans, in response to inhaled allergens. Female and male BALB/c mice (7-8 weeks) were intranasally challenged with the allergen house dust mite (HDM) for 2 weeks. Bronchoalveolar lavage fluid (BALF) was collected 24 hour after the last HDM challenge from allergen-naïve and HDM-challenged mice (N=10 per group, each sex). In a human study, adult participants were exposed to nebulized (2 min) allergens (based on individual sensitivity), BALF was obtained after 24 hour (N=5 each female and male). The BALF samples were examined in immunoblots for the abundance of 10 proteins shown to increase in response to allergen in both murine and human BALF, selected from proteomics studies. We showed significant sex-bias in allergen-driven increase in five out of the 10 selected proteins. Of these, increase in eosinophil peroxidase (EPX) was significantly higher in females compared to males, in both mice and human BALF. We also showed specific sex-related differences between murine and human samples. For example, allergen-driven increase in S100A8 and S100A9 was significantly higher in BALF of females compared to males in mice, but significantly higher in males compared to females in humans. Overall, this study provides sex-specific protein biomarkers that are enhanced in response to allergen in murine and human lungs, informing and motivating translational research in allergic asthma.

## Introduction

Impact of biological sex in disease severity and response to therapy in allergen-mediated asthma is well documented, wherein adult females exhibit higher prevalence and severity compared to males (1, 2). Despite a clear sex disparity in asthma progression, severity and response to therapy, sex-related differences in allergen-mediated protein biomarkers are rarely reported from either preclinical research using animal models or from human studies. Thus, in this study we examined sex-related differences in specific proteins that are enhanced in the lungs in response to allergen, from both mice and humans.

We had previously defined allergen-mediated changes in secreted protein profile (secretome) from bronchoalveolar lavage fluid (BALF) of adult mice and humans (3, 4). These studies did not include sex as a biological variable. A comparative analysis of these previously described lung secretomes (3, 4) identified nineteen proteins that were increased following allergen challenge in both human and mice BALF. Therefore, we selected the top 10 most significantly enhanced proteins for independent examination in this study. These top 10 proteins that increased following allergen challenge in both human and mice BALF were S100A9, coronin1A or TACO (Tryptophan Aspartate-Containing Coat Protein), eosinophil peroxidase (EPX), glycosyl-phosphatidylinositol-specific phospholipase D (GPI-PLD), properdin, CD5 antigen-like protein (CD5L), heterogeneous nuclear ribonucleoprotein U (hnRNPU), lipopolysaccharide-binding protein (LBP), S100A8 and calponin. In this study, the abundance of these selected 10 proteins were independently examined for allergen-mediated alteration in both murine and human BALF, and sex-disaggregated data analysis was used to determine if there was any sex bias in the increase of these proteins following allergen challenge. Protein changes driven by inhaled allergen exposures were examined in BALF samples obtained from a house dust mite (HDM)-challenged murine model of airway inflammation and from human participants exposed to inhaled allergen.

Findings in this study detail sex dimorphism in proteins that are increased in the lungs of mice and humans in response to inhaled allergen. We provide sex-specific protein biomarkers for allergen response, those that are increased similarly and uniquely in murine and human lungs. The results reported in this study will be valuable for translational research in allergic asthma, motivating attention to sex as a biological variable in study design and interpretation.

## Methods

The HDM-challenged mouse model used in this study was similar to that in our previous study defining changes in the lung secretome, in response to the clinically relevant allergen HDM (3). Briefly, adult (7-8 weeks) female and male BALB/c mice were challenged with nasal installation of 35 µL of 0.7 mg/mL of HDM in saline, for 2 weeks. HDM was administered once daily for five consecutive days per week. BALF was collected 24 hour (h) after the last HDM challenge from allergen-naïve (N=10 females and N=10 males) and HDM-challenged (N=10 females and N=10 males) mice. BALF samples were centrifuged (1200rpm for 10 min), supernatants collected for assessment of selected proteins by western blots, and the cell pellets were used for cell differential counts by cytospin using a modified Wright-Giemsa staining (Hema 3^®^ Stat Pack) as previously described by us (5, 6). Supernatants from the BALF samples were concentrated by acetone precipitation, 1 mL cold acetone was added to 150 µL sample, incubated at -20°C overnight, centrifuged at 10,000 xg at 4°C for 10 min and the protein pellets were used for western blots (7). The murine study was approved by The University of Manitoba Animal Research Ethics Board (protocol number AC11394 (B2018-038)) and compliant with ARRIVE guidelines.

In the human study, adult participants (with informed written consent) were exposed to nebulized (2 min) allergens (birch, grass or HDM) based on individual sensitivity in a skin prick test (**Table 1**). The allergen inhalation dose was determined based on minimal concentration to provoke a 3 mm wheal size in the skin prick test and methacholine PC_20_ percent drop and concentration, as previously described by us (8). This study was approved by The University of British Columbia clinical research ethics board (H14-01119) and the Vancouver Coastal Health Research Institute (V14-01119). Given that the clinical focus was on response to allergen in those known to be already sensitized, participants in this study were all atopic and non-smokers. BALF was obtained 24h after allergen exposure (N=5 females and N=5 males) and concentrated using Amicon Ultra 3kD filters. The human samples were anonymized for this study.

**Table 1:** Demographic of human participants.

Sex	Age	Sensitized Allergen
Male	26	Grass
Male	26	Birch
Male	27	Grass
Male	31	HDM
Male	36	HDM
Female	33	HDM
Female	46	HDM
Female	45	HDM
Female	27	Grass
Female	21	Birch

The BALF samples were resolved on 4-12% protein gels (Invitrogen) and probed with antibodies (Abcam) for the aforementioned 10 selected proteins using western blots. Horseradish peroxidase–linked secondary antibodies (Cell Signaling) and Amersham ECL Select (GE Healthcare) were used for detection. Blots were imaged and densitometry performed using Amersham™ Imager 680 and software version 2.0. Mouse BALF samples were spiked with a human protein, recombinant human granulysin (20 ng per sample), and protein band intensities were normalized to the spiked protein as loading control. Similarly, human BALF samples were spiked with a murine protein, recombinant mouse MCP5 (10 ng per sample), which was used as loading control to normalize protein band intensities. Workflow for western blots and data analysis is summarized in **Supplementary Figure 1**.

Sex-disaggregated data analysis was performed (reporting data in female and male separately) based on the SAGER guidelines (9). Statistical analysis for western blot densitometry was performed using Mann-Whitney U test. Person’s correlation analysis was performed to examine the association between protein abundance and cell counts in BALF. All statistical analyses were performed using GraphPad Prism (version 9.3.1; GraphPad Software).

## Results

Here, we report significant sex-related differences in five out of the 10 selected proteins that were enhanced in the BALF in response to inhaled allergen. Comparing our previously published sex-independent studies of allergen-mediated changes in secreted protein profile (secretome) from adult mice and humans (3, 4), we identified 19 proteins that were increased following allergen challenge in both human and mice BALF. Of these, the top 10 most significantly (p<0.05) enhanced proteins in BALF, in order of magnitude of change, were S100A9, TACO, EPX, GPI-PLD, properdin, CD5L, hnRNPU, LBP, S100A8 and calponin. Therefore, these 10 proteins were selected for independent examination by western blots in BALF obtained following allergen challenge from females and males, mice and humans.

**Figure 1:**
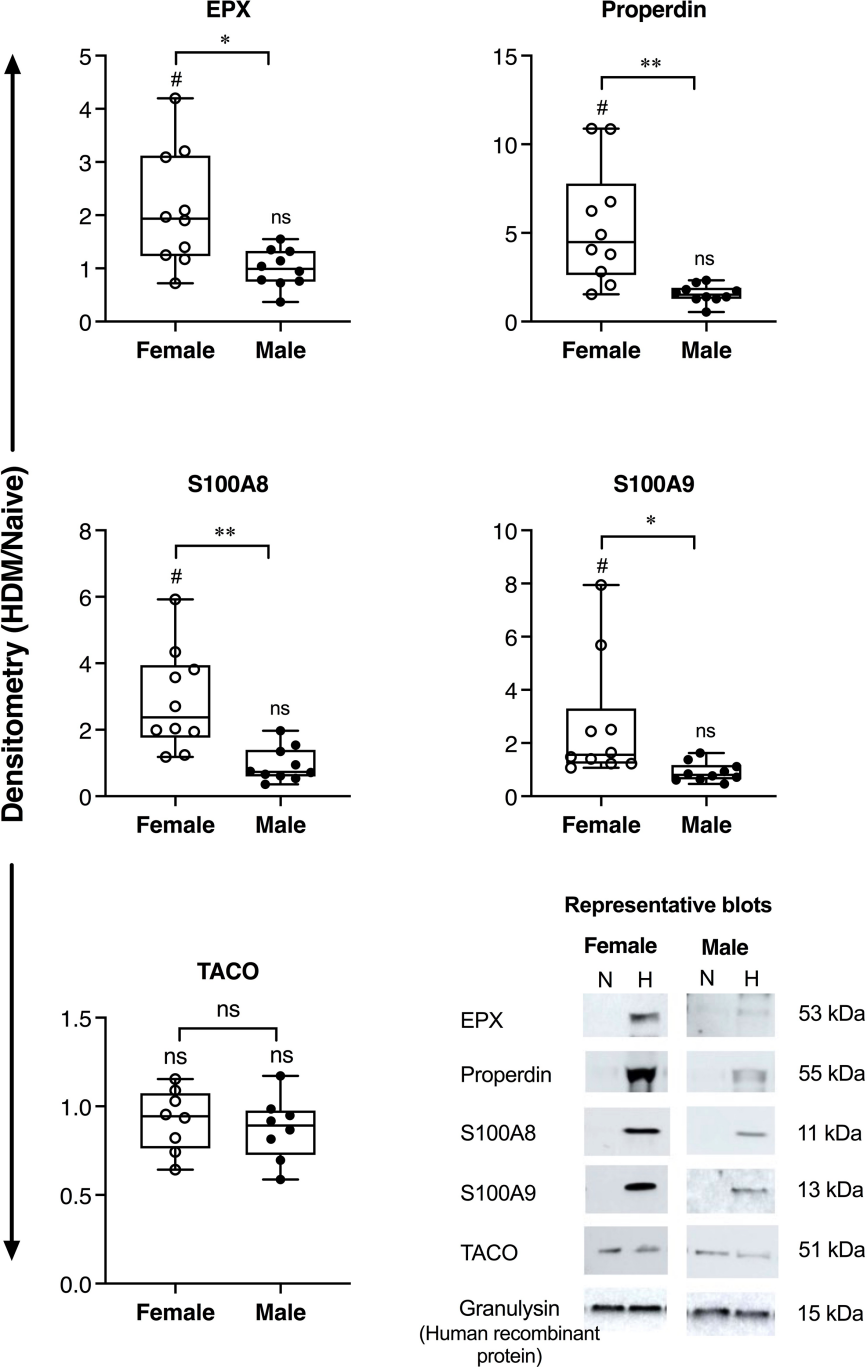
Sex-related differences in protein abundance in mice BALF. Female and male BALB/c mice (N = 10 each) were intranasally challenged with 35 μL (0.7 mg/mL HDM protein extract in saline) per mouse, for 2 weeks. BALF collected from allergen-naïve (N) and HDM-challenged mice (H), 24 h after the last HDM challenge, was concentrated using acetone precipitation and probed for the abundance of selected proteins using western blots. The Y-axis shows protein abundance (band intensity) after normalization with spiked human protein, recombinant human granulysin (20 ng per sample) as loading control. The figure represents densitometry of the ratio of protein abundance in BALF from each HDM-challenged mouse compared to the mean abundance in naïve mice (HDM/naïve). Each dot represents an independent HDM-challenged mouse. Statistical significance was determined by Mann-Whitney U test (*p < 0.05, **p < 0.01, ns, non-significant). Statistical significance of HDM compared to naïve is represented by ^#^which indicates *p*<0.05. Representative blots are also shown for each protein in the bottom panel.

**Figure 2:**
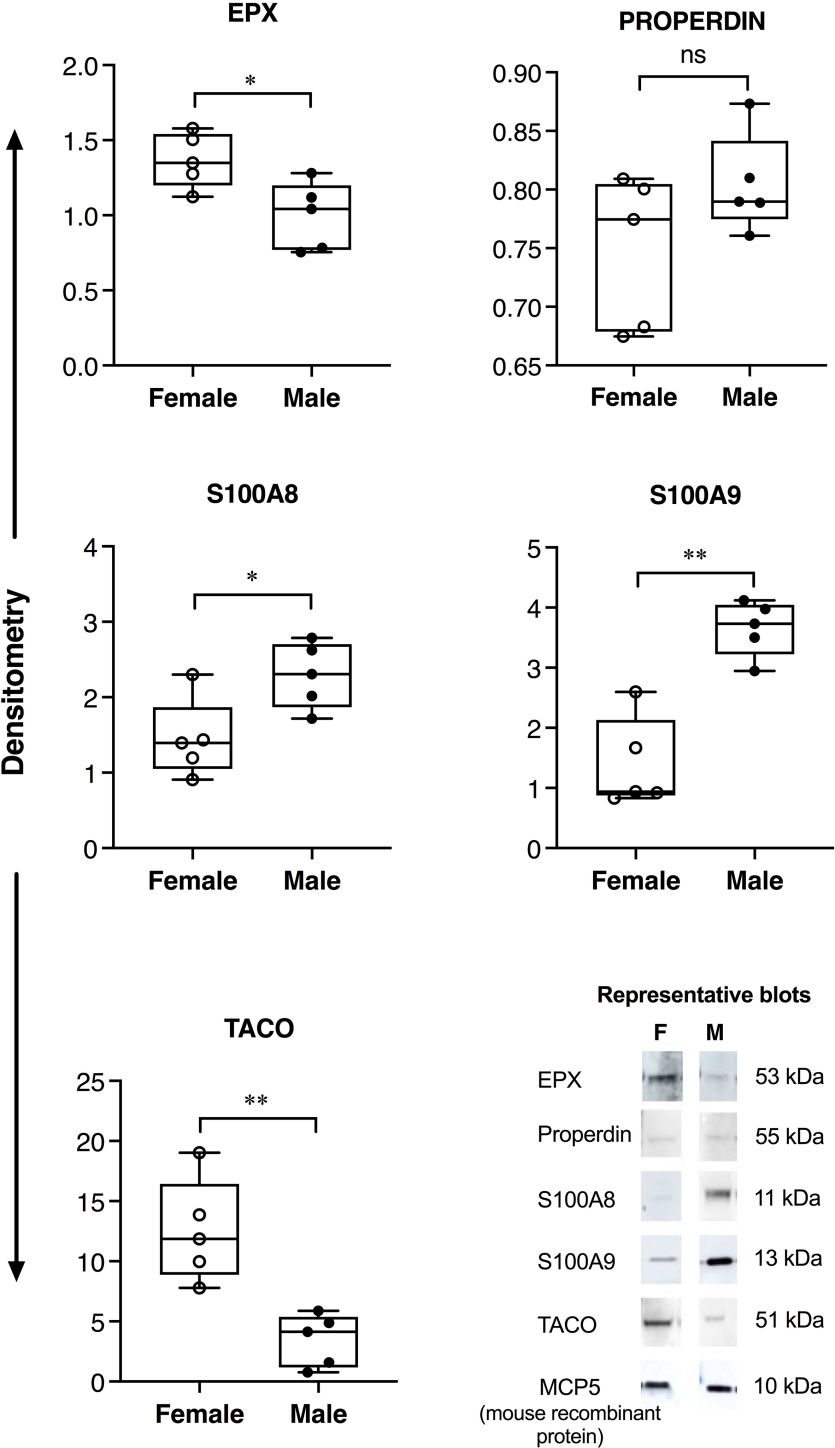
Sex-related differences in protein abundance in human BALF. Adult female (N=5) and male participants (N=5) were exposed to nebulized inhalation (2 min) with allergens (birch, grass or HDM, based on individual sensitization), and BALF collected 24h after challenge. The samples were concentrated using Amicon Ultra 3kD filters and independently probed for the abundance of selected proteins using western blots. The figure represents densitometry analyses where each dot represents BALF sample from an independent participant. Y-axis shows protein abundance (band intensity) normalized to spiked murine protein, recombinant mouse MCP5 (10 ng per sample) as loading control. Statistical significance was determined by Mann-Whitney U test (*p < 0.05, **p < 0.01, ns, non-significant). Representative blots are also shown for each protein in the bottom panel.

Allergen-driven increase in the abundance of BALF proteins were significantly higher in female BALB/c mice for EPX (>2-fold), properdin (~5-fold), S100A8 and S100A9 (>3-fold), compared to males (**Figure 1**). Similarly, EPX abundance was significantly higher in females compared to males, in human BALF in response to inhaled allergen exposure (**Figure 2**). EPX was the only protein that was significantly higher in females compared to males in both mice and humans. Contrary to mice, the increase in S100A8 and S100A9 proteins were significantly higher in male BALF compared to females, in humans (**Figure 2**). In addition, we also identified proteins that showed sex-related differences uniquely in mice and humans. For example, allergen-driven increase in properdin abundance in the BALF was significantly higher in female mice compared to males, but not in humans (**Figures 1** and **2** respectively). Whereas, TACO was significantly higher in female BALF compared to males, only in humans after inhaled allergen challenge (**Figure 2**).

**Figure 3:**
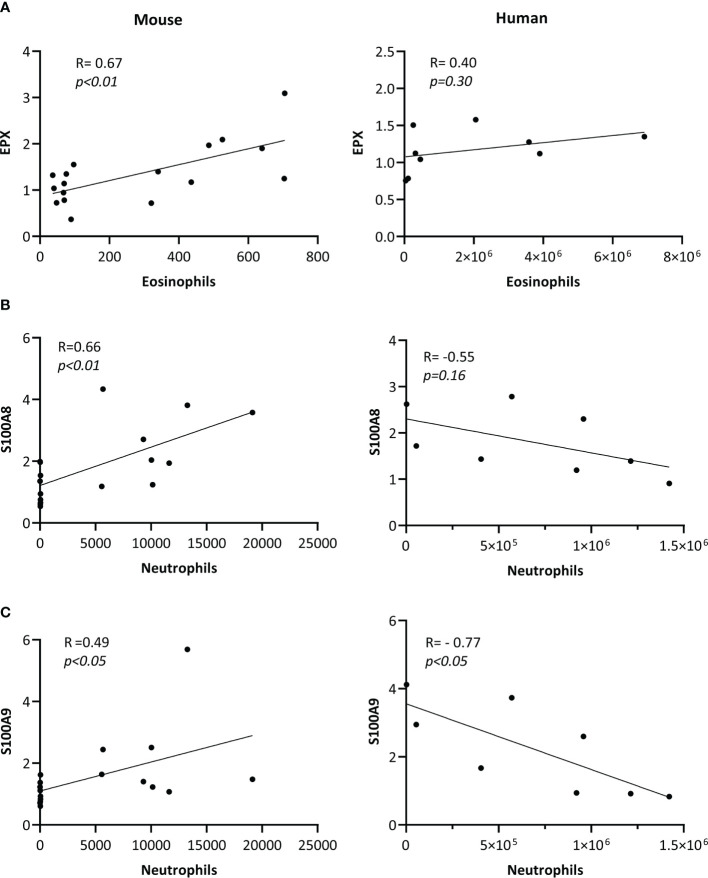
Correlation of protein abundance with cell counts in BALF. BALF samples obtained from allergen-naïve (N = 10 female and N = 10 male) and HDM-challenged (N = 10 female and N = 10 male) BALB/c mice, and from adult human female (N = 4) and male (N = 4) participants exposed to nebulized inhalation (2 min) with allergens based on individual sensitization, were used to assess the abundance of **(A)** EPX, **(B)** S100A8 and **(C)** S100A9 proteins by western blots. Pearson’s correlation analysis was performed to determine the association between HDM-driven increases (HDM/naïve) in each protein with cell counts, as indicated in the figure, for murine BALF. Pearson’s correlation analysis was performed to determine the association between the abundance of each protein with cell counts, as shown in the figure, for human BALF samples. Correlation analyses show associations between **(A)** EPX with eosinophils/mL BALF counts, **(B)** S100A8 with neutrophils/mL of BALF, and **(C)** S100A9 with neutrophil/mL of BALF, and *p*< 0.05 was considered to be statistically significant.

As EPX is an eosinophil-derived protein (10), we examined the association between HDM-driven increase in the abundance of EPX (western blot densitometry; HDM/naïve) with eosinophil counts (HDM/naïve), in murine BALF. In mice, there was a significant (*p*<0.01) positive correlation between EPX abundance and eosinophil counts in BALF (**Figure 3A**). However, abundance of EPX did not show significant correlation with eosinophil counts in human BALF (**Figure 3A**). As S100A8 and A9 proteins are neutrophil-associated (11, 12), we performed correlation analyses between these proteins and neutrophil counts in BALF. HDM-driven increase in S100A8 and S100A9 levels in murine BALF showed a significant positive correlation with neutrophil counts (**Figures 3B, C** respectively). Although there was no significant correlation between S100A8 and neutrophil counts, there was a significant negative correlation between S100A9 abundance and neutrophils in human BALF (**Figure 3C**).

## Discussion

To our knowledge, this is the first study to compare and contrast sex dimorphism of specific proteins in response to an allergen in the lungs of mice and humans. In this study, we report significant sex-related differences in five out of the 10 selected proteins that were enhanced in the BALF in response to airway allergen challenge. We demonstrate that allergen-driven increase in EPX is significantly higher in females compared to males, in both mice and human BALF. EPX is an eosinophil-derived cationic protein and a marker of eosinophil activation and degranulation (10). Consistent with this, we show a significant positive correlation between allergen-driven enhancements of EPX abundance with eosinophil counts in murine BALF. However, we did not observe a significant correlation between EPX abundance and eosinophil counts in human BALF, which is most likely due to the limited number of human samples in this study. Nevertheless, our results are corroborated by clinical evidence showing that the severity of allergen-mediated eosinophilic airway inflammation is higher in females compared to males (1). Our results suggest that EPX is a female-biased biomarker of allergen response which reflects common biology in humans and mice. Airway epithelial cells and eosinophils express estrogen receptors, and it has been shown that estradiol can promote degranulation of eosinophils, whereas testosterone decreases eosinophil recruitment to the lungs in allergic airway inflammation (13). Therefore, it is likely that the higher abundance of EPX in females may be due to the differential action of sex steroids in eosinophilic airway inflammation, which needs to be further elucidated. Our findings indicate that EPX will be a valuable biomarker, especially for translational research examining response to candidate therapeutics in females for allergic asthma.

We also demonstrate specific differences in sex dimorphism of proteins that are enhanced in BALF following inhaled allergen exposures between mice and humans. Our results show that allergen-mediated enhancements of S100A8 and S100A9 proteins are significantly higher in BALF of female mice compared to males, but this pattern is reversed in humans. S100A8 and S100A9 are alarmins implicated in various chronic inflammatory conditions, including asthma (14). S100A8 and S100A9 promote allergic airway inflammation, in particular neutrophilic inflammation (15, 16). Similar to protein abundance, we report discordant associations between S100A8/A9 abundance and neutrophil counts in mice and human BALF. We demonstrate a significant positive correlation between allergen-driven increases in S100A8 and S100A9 proteins with neutrophil counts in the murine BALF, whereas there was a significant negative correlation between S100A9 abundance and neutrophil counts in humans BALF. Although S100A8 and S100A9 proteins are associated with severe uncontrolled asthma, there are conflicting results as their function have been demonstrated both as a mediator and suppressor of Th2-skewed airway inflammation in animal models (11, 12). A recent study has shown that S100A8/A9 suppress neutrophil apoptosis and elevate cytokines associated with neutrophilic inflammation by engaging TLR4 and RAGE receptors (15). Interestingly, TLR4-mediated signaling and downstream inflammatory responses are increased in the presence of testosterone (17). This is corroborated by our results showing higher enhancement of S100A8/A9 in males compared to females in human BALF in response to allergens, although not in mice. Nevertheless, results in this study clearly demonstrate sex bias in the enhancement of both S100A8 and S100A9 proteins in response to airway allergen challenge, but with opposite sex-related difference in mice and humans. It should be noted that the animal model reflects effects of repeated exposure to allergen, while the human exposure represents an acute exposure in sensitized individuals. These differences in allergen exposures could contribute to the opposite effects observed for S100A8/A9 protein changes between mice and human BALF.

We also show that allergen-mediated increase in Properdin, a regulator of the complement cascade in airway inflammation, is significantly higher in female mice compared to males, but not in humans. Properdin levels are higher in the BALF of patients with allergic asthma and elevated in the BALF of OVA-sensitive mice (18, 19). Properdin has been suggested to promote Th2- and Th17-mediated allergic airway inflammation, and is associated with eosinophilic inflammation (18). We show that both EPX and Properdin, markers of eosinophilic airway inflammation, are higher in females compared to males in the mouse model, which corroborates evidence that eosinophilic airway inflammation is higher in females compared to males (1).

In contrast to Properdin, we show that allergen-mediated increase in TACO or coronin-1A is significantly higher in BALF from human females compared to males, but this is not the case in mice. TACO is expressed in mammalian leukocytes and a recent study reported that it is altered in the sputum of patients with airway inflammation and mucous hypersecretion, for example it is suppressed in COPD, enhanced in chronic bronchitis, and altered with variable differences in asthma (20). The biological relevance of TACO in the context of airway inflammation and asthma is yet to be determined. Overall, the discrepancies in the abundance of S100A8/A9, Properdin and TACO between mice and human reported here demonstrate that sex is a differential effect modifier of certain allergen-mediated proteins in the lungs of mice compared to humans. These results underscore the importance of selecting protein markers in mouse models that can be translatable to human studies.

A limitation in this study is the small sample size of human BALF samples, and that the human BALF samples were only from participants with atopy. Despite the small sample size we have demonstrated statistically significant sex-related differences in allergen-mediated increase of specific proteins in the human BALF. The mouse model used in this study represents a model of robust airway inflammation with airway hyperresponsiveness, but preceding significant fibrosis (5). Therefore, proteins defined in this study will be primarily relevant in the context of allergen-mediated airway inflammation.

Overall, the findings in this study demonstrate a clear sex-bias in specific proteins that are increased in the BALF in response to inhaled allergen challenge, in mice and humans. Proteins reported in this study will be useful as biomarkers for integrating sex as a biological variable both in preclinical studies using mouse models and in human studies, relevant to allergic asthma research. Findings reported here provide clues to biological processes that may determine intervention strategies aimed to alleviate disparate asthma severity in adult females and males.

## Data Availability Statement

The original contributions presented in the study are included in the article/**Supplementary Material**. Further inquiries can be directed to the corresponding author.

## Ethics Statement

The studies involving human participants were reviewed and approved by The University of British Columbia clinical research ethics board (H14-01119) and the Vancouver Coastal Health Research Institute (V14-01119). The patients/participants provided their written informed consent to participate in this study. The animal study was reviewed and approved by The University of Manitoba Animal Research Ethics Board [protocol number AC11394 (B2018-038)].

## Author Contributions

MH and DM (shared first authors) performed most of the experiments and data analyses. MH wrote the manuscript and contributed to the development of the scientific concepts. HP performed the animal model experiment with DM. VS performed the bioinformatics analysis. DM-B and AB recruited human participants and assisted in collection of human samples. AH provided significant intellectual input in the development of this study and extensively edited the manuscript. CC was the senior lead for the human exposure study, performed the bronchoscopy and sample collection, provided extensive intellectual input and extensively edited the manuscript. NM conceived and directly supervised the study, is the principal investigator for funding in this study, and extensively edited the manuscript. All authors reviewed and edited the manuscript. All authors contributed to the article and approved the submitted version.

## Funding

This study was supported by funding from the Canadian Institutes of Health Research (grant numbers SVB-158629 and GS2-171363) and Manitoba Workers Compensation Board (RWIP17-03). HP was supported by studentships from Research Manitoba, Asthma Canada and the AllerGen Network. AH is supported by the Canada Research Chairs (CRC) Program. CC is supported by the CRC program, the Astra-Zeneca Chair in Occupational and Environmental Lung Disease, and the Michael Smith Foundation for Health Research.

## Conflict of Interest

The authors declare that the research was conducted in the absence of any commercial or financial relationships that could be construed as a potential conflict of interest.

## Publisher’s Note

All claims expressed in this article are solely those of the authors and do not necessarily represent those of their affiliated organizations, or those of the publisher, the editors and the reviewers. Any product that may be evaluated in this article, or claim that may be made by its manufacturer, is not guaranteed or endorsed by the publisher.
