# Supplementary material for: Sex Dimorphism of Allergen-Induced Secreted Proteins in Murine and Human Lungs
Source: Front Immunol. 2022 Jun 28;13:923986. doi: 10.3389/fimmu.2022.923986 (PMC9273854; doi:10.3389/fimmu.2022.923986)

Brief Research Report

Sex dimorphism of allergen-induced secreted proteins in murine and human lungs

Supplementary Information

Supplementary Figure 1: Flow chart of workflow for western blots and data analysis.


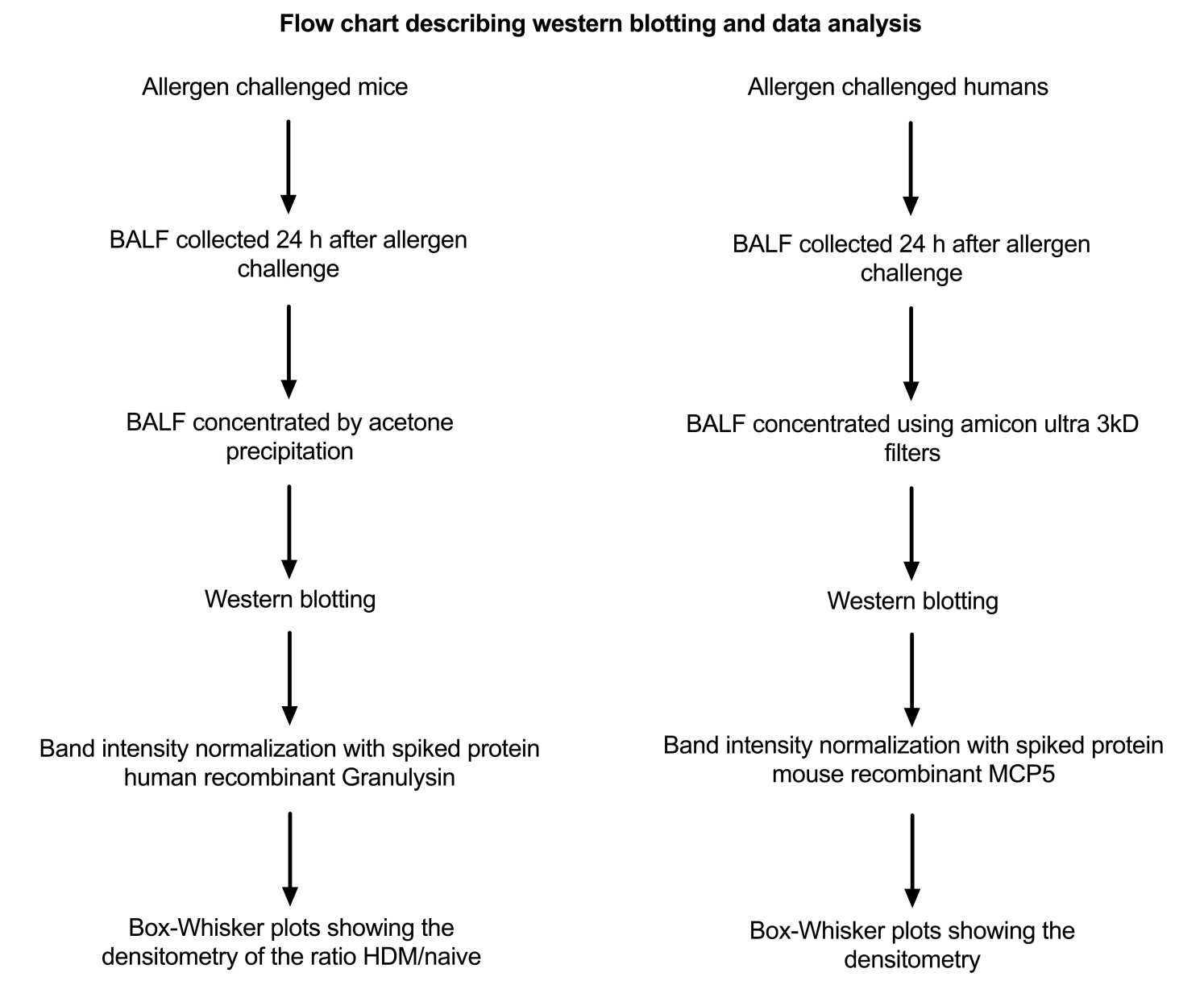

Supplement: Supplementary file 1 [file DataSheet_1.docx]
